# Draft Genome Sequence of *Acidithiobacillus* sp. Strain SH, a Marine Acidophilic Sulfur-Oxidizing Bacterium

**DOI:** 10.1128/genomeA.01603-17

**Published:** 2018-02-08

**Authors:** Kazuo Kamimura, Sultana Sharmin, Eriko Yoshino, Mirai Tokuhisa, Tadayoshi Kanao

**Affiliations:** a Graduate School of Environmental and Life Science, Okayama University, Okayama, Japan

## Abstract

We announce here the genome sequence of a marine acidophilic sulfur-oxidizing bacterium, *Acidithiobacillus* sp. strain SH. The bacterium has potential for use in bioleaching of sulfide ores from seawater and contains a noble gene for thiosulfate quinone oxidoreductase in addition to specific genes for the oxidation of reduced inorganic sulfur compounds.

## GENOME ANNOUNCEMENT

The genus *Acidithiobacillus* comprises a group of acidophilic chemolithoautotrophic bacteria capable of oxidizing reduced inorganic sulfur compounds (RISCs), thereby contributing to the bioleaching of ores. Halophilic acidophiles have gained increasing interest because of their importance in bioleaching operations in salt-containing environments (1–3). *Acidithiobacillus* sp. strain SH (NBRC 101132) is a Gram-negative acidophilic, mesophilic, and chemolithoautotrophic marine bacterium with sodium chloride-stimulated sulfur-, sulfite-, and thiosulfate-oxidizing activities (4, 5). A noble thiosulfate quinone oxidoreductase (TQO) has been purified from strain SH grown on thiosulfate-containing medium (6). Because analysis of peptide fragments produced by the in-gel trypsin digestion of TQO revealed no protein having high homology with TQO, we tried to determine the gene for TQO in the draft genome sequence of strain SH.

Whole-genome sequencing was performed using a Roche FLX Titanium genome sequencer (for 8-kb-long paired-end sequencing) and FLX+ technology (for shotgun sequencing) provided by Operon Biotechnologies (Tokyo, Japan). A shotgun library was prepared and sequenced, generating 245,865 reads in 161.68 Mb of sequencing data. An 8-kb-long paired-end library was also sequenced, generating 226,288 reads in 36.18 Mb of sequencing data. An average coverage of 66.8× of the genome was obtained. Coassembly of the results from both shotgun and paired-end sequencing was performed by Newbler version 2.6. The genes were annotated using the NCBI Prokaryotic Genome Annotation Pipeline version 4.1 and GeneMarkS+.

The draft genome of strain SH is approximately 2.9 Mb in size, distributed in 65 contigs with an average G+C content of 54.3%. Of 2,978 genes, 2,844 were predicted to be protein-coding genes and 52 were RNA genes. RNA genes partitioned into one 5S-16S-23S rRNA operon and 45 tRNAs. Genome analyses showed the presence of genes for carboxysome and carbon dioxide fixation via the Calvin-Benson-Bassham cycle. The following genes for RISC metabolism were found: two gene clusters encoding the sulfur oxidation complex SOX (*soxYZB*-*hyp*-*resB*-*soxXA* and *soxXYZA*-*hyp*-*soxB*), a tetrathionate hydrolase gene (*tetH*), two thiosulfate quinone oxidoreductase genes (*doxD*), and a sulfide quinone reductase gene (*sqr*). These genes were previously identified in *Acidithiobacillus caldus* (7), *A. thiooxidans* (8, 9), and *A. albertensis* (10). Although strain SH has been identified as *A. thiooxidans* based on the sequence similarity (99.3%) with the 16S rRNA gene of *A. thiooxidans* ATCC 19377 (4), the average nucleotide identity displayed a low degree of similarity (82.3%) with the genome of *A. thiooxidans* ATCC 19377. Therefore, we use the undefined species organism name, *Acidithiobacillus* sp. strain SH, for this genome until this genome can be better evaluated.

The draft genome sequence of strain SH enabled us to determine a gene encoding the noble TQO, providing further insights into the genomic diversity of members of the genus *Acidithiobacillus*, and contributes to a better understanding of the mechanism for RISC metabolism in sulfur-oxidizing prokaryotes.

### Accession number(s).

This whole-genome shotgun project has been deposited at DDBJ/ENA/GenBank under the accession number MXAV00000000. The version described in this paper is the first version, MXAV01000000.

## References

[1] Huber H, Stetter KO 1989 *Thiobacillus prosperus* sp. nov., represents a new group of halotolerant metal-mobilizing bacteria isolated from a marine geothermal field. Arch Microbiol 151:479–485. doi:10.1007/BF00454862.

[2] Davis-Belmar CS, Nicolle JLC, Norris PR 2008 Ferrous iron oxidation and leaching of copper ore with halotolerant bacteria in ore columns. Hydrometallurgy 94:144–147. doi:10.1016/j.hydromet.2008.05.030.

[3] Khaleque HN, Ramsay JP, Murphy RJ, Kaksonen AH, Boxall NJ, Watkin ELJ 2017 Draft genome sequence of the acidophilic, halotolerant, and iron/sulfur-oxidizing *Acidihalobacter prosperus* DSM 14174 (strain V6). Genome Announc 5(3):e01469-16. doi:10.1128/genomeA.01469-16. 28104654 PMC5255921

[4] Kamimura K, Higashino E, Moriya S, Sugio T 2003 Marine acidophilic sulfur-oxidizing bacterium requiring salts for the oxidation of reduced inorganic sulfur compounds. Extremophiles 7:95–99. doi:10.1007/s00792-004-0420-5. 12664261

[5] Kamimura K, Higashino E, Kanao T, Sugio T 2005 Effects of inhibitors and NaCl on the oxidation of reduced inorganic sulfur compounds by a marine acidophilic, sulfur-oxidizing bacterium, *Acidithiobacillus thiooxidans* strain SH. Extremophiles 9:45–51. doi:10.1007/s00792-004-0420-5. 15375674

[6] Sharmin S, Yoshino E, Kanao T, Kamimura K 2016 Characterization of a novel thiosulfate dehydrogenase from a marine acidophilic sulfur-oxidizing bacterium, *Acidithiobacillus thiooxidans* strain SH. Biosci Biotechnol Biochem 80:273–278. doi:10.1080/09168451.2015.1088377. 26393925

[7] Valdes J, Quatrini R, Hallberg K, Dopson M, Valenzuela PDT, Holmes DS 2009 Draft genome sequence of the extremely acidophilic bacterium *Acidithiobacillus caldus* ATCC 51756 reveals metabolic versatility in the genus *Acidithiobacillus*. J Bacteriol 191:5877–5878. doi:10.1128/JB.00843-09. 19617360 PMC2737959

[8] Valdes J, Ossandon F, Quatrini R, Dopson M, Holmes DS 2011 Draft genome sequence of the extremely acidophilic biomining bacterium *Acidithiobacillus thiooxidans* ATCC 19377 provides insights into the evolution of the *Acidithiobacillus* genus. J Bacteriol 193:7003–7004. doi:10.1128/JB.06281-11. 22123759 PMC3232857

[9] Yin H, Zhang X, Li X, He Z, Liang Y, Guo X, Hu Q, Xiao Y, Cong J, Ma L, Niu J, Liu X 2014 Whole-genome sequencing reveals novel insights into sulfur oxidation in the extremophile *Acidithiobacillus thiooxidans*. BMC Microbiol 14:179. doi:10.1186/1471-2180-14-179. 24993543 PMC4109375

[10] Castro M, Moya-Beltrán A, Covarrubias PC, Gonzalez M, Cardenas JP, Issotta F, Nuñez H, Acuña LG, Encina G, Holmes DS, Johnson DB, Quatrini R 2017 Draft genome sequence of the type strain of the sulfur-oxidizing acidophile, *Acidithiobacillus albertensis* (DSM 14366). Stand Genomic Sci 12:77. doi:10.1186/s40793-017-0282-y. 29255572 PMC5731081

